# KLF2 determines the susceptibility of T cells to immunoregulatory NK cells

**DOI:** 10.21203/rs.3.rs-4921081/v1

**Published:** 2024-08-30

**Authors:** Stephen Waggoner, Andrew Cox, Laura Canaday, Alexander Katko, Harrison Feldman, Kathrynne Warrick, Anastassia Tselikova, Harsha Seelamneni, Krishna Roskin

**Affiliations:** Cincinnati Children’s Hospital Medical Center; Cincinnati Children’s Hospital Medical Center; Cincinnati Children’s Hospital Medical Center; Cincinnati Children’s Hospital Medical Center; Cincinnati Children’s Hospital Medical Center; Cincinnati Children’s Hospital Medical Center; UCLA; Cincinnati Children’s Hospital Medical Center; Cincinnati Children’s Hospital Medical Center

**Keywords:** migration, immune suppression, vaccine, innate lymphoid cell

## Abstract

Natural killer (NK) cells suppress cellular and humoral immune responses via killing of T cells, resulting in diminished vaccine responses in mice and humans. Efforts to overcome this roadblock and achieve optimal immunity require an improved understanding of the molecular mediators facilitating NK cell-targeting of discrete subsets of CD4 T cells. We employed single-cell forensic victimology and CRISPR-Cas9 editing to delineate a transcriptional program uniquely responsible for the susceptibility of a subpopulation of CD4 T cells to perforin-dependent immunoregulation by NK cells. The unique vulnerability of these CD4 T cells relative to other subsets of CD4 T cells was not associated with a pattern of NK-cell-receptor ligand expression that would favor activation of NK cells. Instead, susceptible CD4 T cells were skewed toward follicular helper T cell (Tfh) differentiation and exhibited intermediate expression of Klf2 and a related suite of KLF2-target genes (e.g. S1pr1) involved in cell migration and spatial positioning. NK-cell dependent suppression of the subset of Tfh exhibiting intermediate expression of KLF2 and S1PR1 was confirmed with single-cell proteomics. CRISPR targeting of KLF2 in CD4 T cells prevented suppression by NK cells. Thus, KLF2 regulation of spatial positioning of T cells is a key determinant of NK-cell immunoregulatory function and a possible target for strategies to enhance vaccine efficacy.

## Introduction

Natural killer cells govern vaccine responses in mice and humans. Clinical studies reveal an inverse relationship between vaccine efficacy and NK cell activity following immunization with protein (hepatitis B virus, RECOMBIVAX HB), polysaccharide conjugate (pneumococcal PCV13), protein/adenovirus prime-boost (RTS,S malaria), and live attenuated virus (yellow fever-17D) vaccines.^1–4^ Human NK cell cytolytic function is a hindrance specifically for T-cell dependent vaccines^1^ and generation of HIV-specific broadly neutralizing antibodies.^5^ These observations dovetail with mechanistic studies in mice by our group and others, which collectively reveal an early killing of a fraction of activated CD4 T cells by NK cells after immunization or infection that undermines subsequent humoral immune responses.^6–9^ While receptors on NK cells such as 2B4 and NKG2A prevent exaggerated suppression of T cells that could undermine immunity,^10–13^ the mechanisms that prompt the targeted killing of a discrete subset of activated CD4 T cells remain ill-defined. Development of an effective and narrowly focused translational strategy to enhance vaccine efficacy by subverting immunoregulatory NK cells requires a detailed understanding of the mechanisms underlying this selectivity of this activity against certain T cells.^14^

### NK cells target emerging Tfh during clonal expansion.

**Figure 1:**
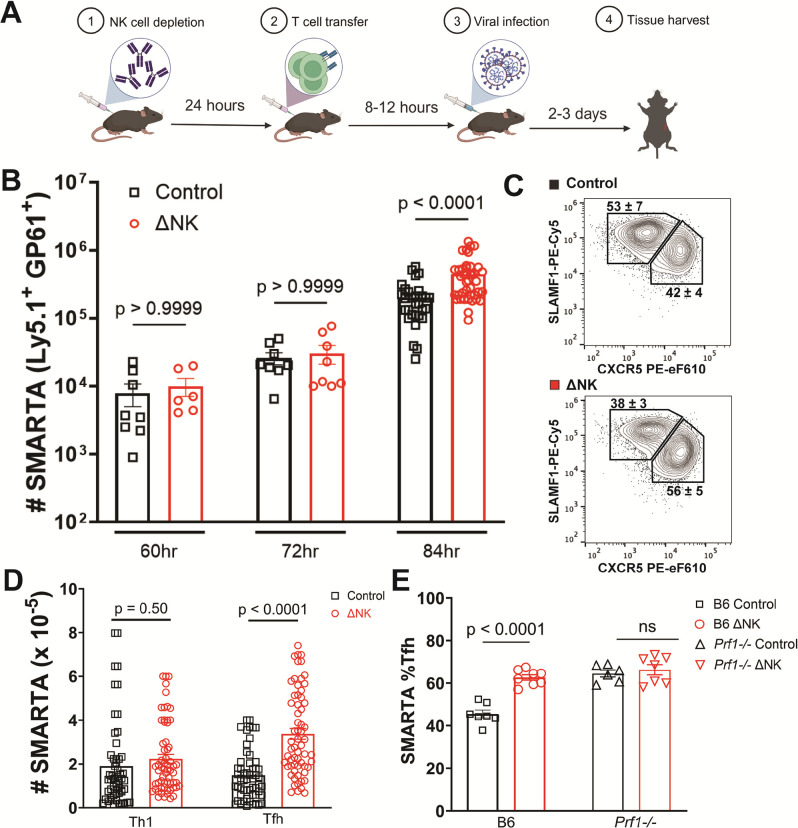
NK cells suppress early expansion and follicular differentiation of antiviral CD4 T cells. **A)** experimental layout: C57BL/6 mice were depleted of NK cells with 25 μg of anti-NK1.1 antibody or control mouse IgG2a prior to intravenous infusion of 5×10^5^ SMARTA Ly5.1^+^ CD4 T cells followed by intraperitoneal infection with 5×10^4^ pfu of the Armstrong strain of LCMV. At various time points after infection I-A^b^:LCMV-GP_66–77_-Tetramer+ CD45.1^+^ SMARTA were quantified in spleen by flow cytometry to determine **B)** mean (± s.e.m.) total number of splenic SMARTA T cells, **C)** representative gating scheme, **D)** number of SMARTA with Tfh- (CXCR5^+^ SLAMF1^lo^) or Th1-phenotype (CXCR5^lo^ SLAMF1^hi^). Data from 8–32 recipient mice/group compiled from 2–8 independent experiments. **E)** mean (± s.e.m.) total number of splenic SMARTA T cells when Prf −/− animals are used as recipients. Statistical differences between comparator groups at each time point determined by Student’s T test.

We undertook a forensic victimology approach to gain insights into the population of T cells susceptible to and regulated by NK cells. We used lymphocytic choriomeningitis virus (LCMV) infection as a robust trigger of NK-cell regulation of T cells, a phenomenon more broadly observed with multiple viral and bacterial pathogens as well as vaccine regimens.^14^ Transgenic congenically-marked LCMV GP66–80-specific CD4 T cells (denoted SMARTA) were seeded at in C57BL/6 mice prior to antibody-mediated selective depletion of NK cells (ΔNK or isotype Control treatment)^10^ and infection with the Armstrong strain of LCMV (Figure 1A). A statistically significant increase in the number of SMARTA T cells was first seen in the NK-depleted animals 84 hours post-infection, where SMARTA underwent 44-fold expansion (5–6 cell divisions) in the absence of NK cells but only a 25-fold expansion in the presence of NK cells (Figure 1B). Twelve hours earlier (72h post infection (p.i.)), SMARTA cells had only undergone 3-fold expansion (1–2 cell divisions) that was statistically similar between control and NK cell-depleted mice, suggesting NK cell regulation occurs between the first and fifth cycles of cell division during proliferative expansion. Using CXCR5 and SLAMF1 as markers of CD4 T cell differentiation^15^ after the first cell division in this model, we observed that NK cells predominately restrained the development of a T follicular helper (Tfh) phenotype (CXCR5^high^ SLAMF1^low^) but not that of T helper 1 (Th1) like cells (CXCR5^low^ SLAMF1^high^) (Figure 1C,D). Genetic ablation of perforin (Prf1^−/−^) or depletion of NK cells both biased the differentiation of SMARTA toward Tfh lineage to similar extents without evidence that concomitant ablation of NK cells and perforin exerted additive effects (Figure 1E), confirming that early perforin-dependent mechanisms underlie NK cell suppression of developing CD4 T cell responses.^8^

### Selective targeting of a subset of developing Tfh

**Figure 2:**
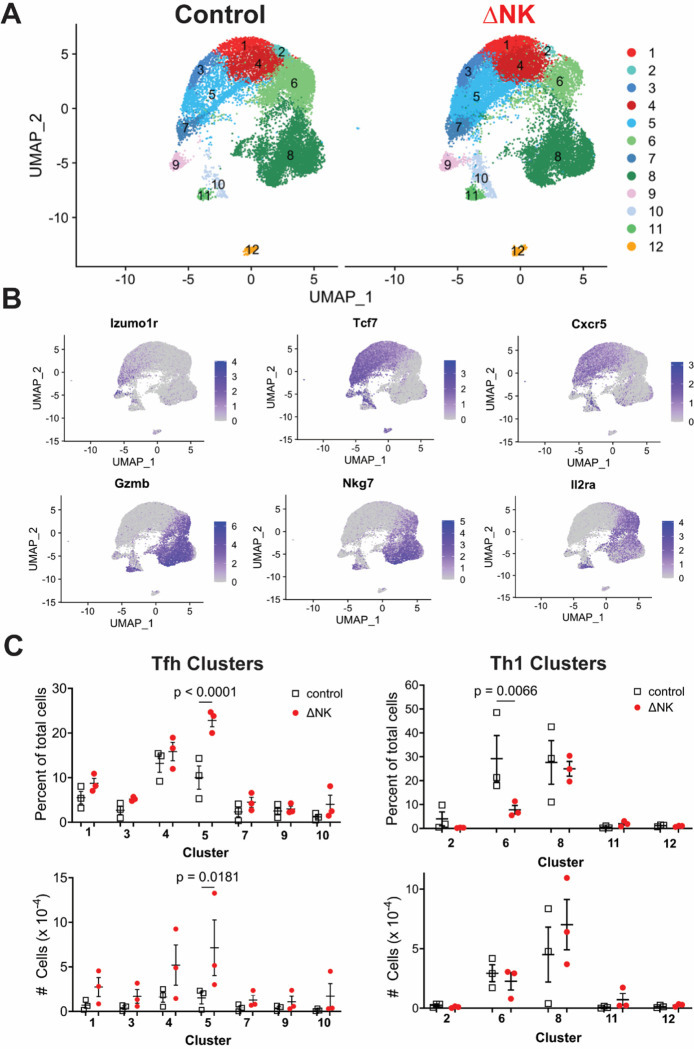
Single cell transcriptomic analysis reveals an unique cluster of emerging Tfh targeted by NK cells. SMARTA CD4 T cells were transferred into NK cell-replete or -depleted C57BL/6 mice prior to infection as described in Figure 1. At day 3.5 post-infection, CD4 T cells were enriched from spleen by negative selection (Miltenyi) and CD45.1^+^ TCR-Vα2^+^ CD4+ T cells were FACS sorted from three mice per condition. These cells were prepared via the 10X genomics platform for single cell sequencing with 5’ chemistry and sequenced at depth of 25,000 reads per cell. 11,524 and 21,234 cells in the control and NK cell-depleted groups, respectively passed QC. **A)** Resulting UMAP distribution and clustering of SMARTA CD4 T cells. **B)** Feature plot of the transcript levels of genes defining either Tfh (*Izumo1r*, *Tcf7*, and *Cxcr5*) or Th1 (*Gzmb*, *Nkg7*, and *Il2ra*). **C)** Frequency and number (mean ± s.e.m.) of cells falling into the 12 clusters grouped by similarity into Tfh and Th1 for each recipient animal. Statistical analyses performed with one-way ANOVA, only showing p≤0.05 for clarity.

We next performed single-cell transcriptomic analysis of isolated SMARTA (**Extended Data Figure 1A**) at 84 hours p.i. to determine characteristics of the subpopulation(s) affected by NK cells (Figure 2A). Notably, no subsets were present exclusively in the absence of NK cells, suggesting that susceptibility to immunoregulation is not a uniform feature of a discrete subpopulation of cells. Consistent with our flow cytometry data (Figure 1) and past reports,^15^ categorization of the resulting clusters largely dichotomizes the SMARTA into Tfh-like (*Tcf7, Cxcr5*, *Izumo1r*) and Th1-like (*Gzmb, Nkg7, Il2ra*) groups (Figure 2B), which were further divided based on Silhouette Score optimization. A single subpopulation of Tfh-like SMARTA (Cluster 5) exclusively among the twelve subsets identified was significantly reduced in both proportion and number in the presence of NK cells (Figure 2C). One subpopulation of Th1-like cells (Cluster 6) was proportionally increased in the presence of NK cells, although this difference was not borne out in terms of cell number (Figure 2C).

### T-cell susceptibility dissociated from classical NK cell-activating signals.

**Figure 3:**
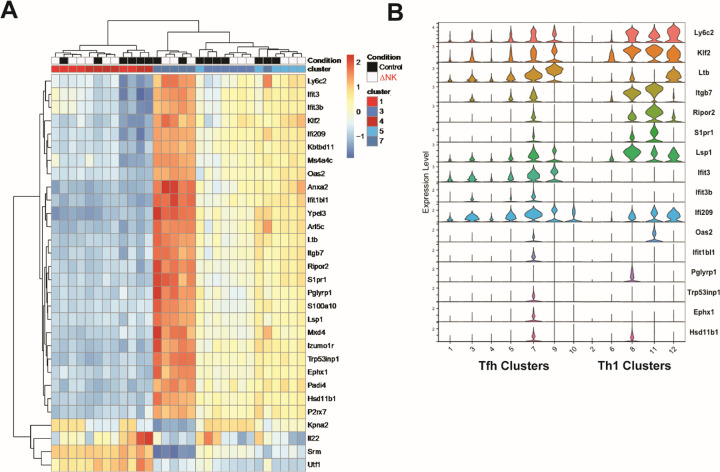
Susceptibility of emerging Tfh to NK-cell suppression linked to intermediate KLF2-driven gene regulatory program. Clusters were collapsed via pseudobulking and analyzed via DESeq2. A) Heatmap of the 30 genes with the highest variance between the Tfh clusters. **B)** Violin plots of the single cell data of the positioning related genes and IFN responsive genes selected by the unbiased DESeq2 analysis.

Activation of cytotoxic functions of NK cells is triggered by an imbalanced engagement of activating over inhibitory receptors.^16^ We hypothesized that Cluster 5 might be targeted due to uniquely low expression of ligands for inhibitory NK cell receptors, high expression of ligands for activating NK cell receptors, or a combination thereof. To mitigate potential dropout artifacts inherent to single-cell sequencing, we performed pseudobulk analysis to query the characteristics of this expanded population. Surprisingly, expression of an array of ligands for NK-cell receptors ranked as intermediate on Cluster 5 in comparison to the other subpopulations of T cells (**Extended Data Figure 1B,C**). Thus, susceptibility of T cells to NK cell regulation is not associated with a pattern of NK cell-receptor ligands that would be predicted to be more favorable for NK cell activation than apparently refractory subpopulations of T cells. Intriguingly, the targeted T cell subset (Cluster 5) exhibited an intermediate pattern of interferon-stimulated gene (ISG) expression (*Lgals3, Ifit3, Ifit3b, Ifi209, Oas2, Kbtbd11, Ms4a4c, Ifitbl1*) relative to other subpopulations (Figure 3A). Given that type I IFN signaling can protect T cells from NK cell cytotoxic activity,^17^ this intermediate ISG response in Cluster 5 may contribute to the greater susceptibility of these cells to NK cell regulation. However, Th1-like clusters (cluster 2, 6, 8, 11, 12) that are refractory to NK-cell suppression generally show an even weaker ISG response than most of the Tfh-like clusters (Figure 3B), suggesting that strength of IFN signaling cannot fully explain this phenomenon.

### Tfh susceptibility to NK cells linked to KLF2 transcriptional program.

The NK-cell susceptible Cluster 5 is defined by an intermediate expression of genes encoding the transcription factor krüpple-like factor 2 (*Klf2*) and a set of KLF2-regulated genes (*Ly6c2, Klf2, Itgb7, S1pr1, Lsp1, Rasa3*) with functions in spatial positioning^18,19^ (Figure 3A). Of note, Th1-like cells characteristically maintain high levels of KLF2 and position within the T cell rich regions of lymphoid tissues, whereas Tfh express low levels of KLF2 to facilitate positioning in B-cell follicles.^20^ Correspondingly, Th1-related clusters 8, 11, and 12 exhibit higher levels of KLF2 expression relative to any of the Tfh-like cell clusters (Figure 3B). Thus, intermediate expression of KLF2 putatively positions the NK cell-susceptible subset of T cells at the interface between T- and B-cell zones, a site where we have previously observed NK cell enrichment at days 2 and 3 of LCMV infection.^21^ We hypothesize that a KLF2-driven transcriptional program in Cluster 5 cells contributes to positional susceptibility to immunoregulatory functions of NK cells.

**Figure 4:**
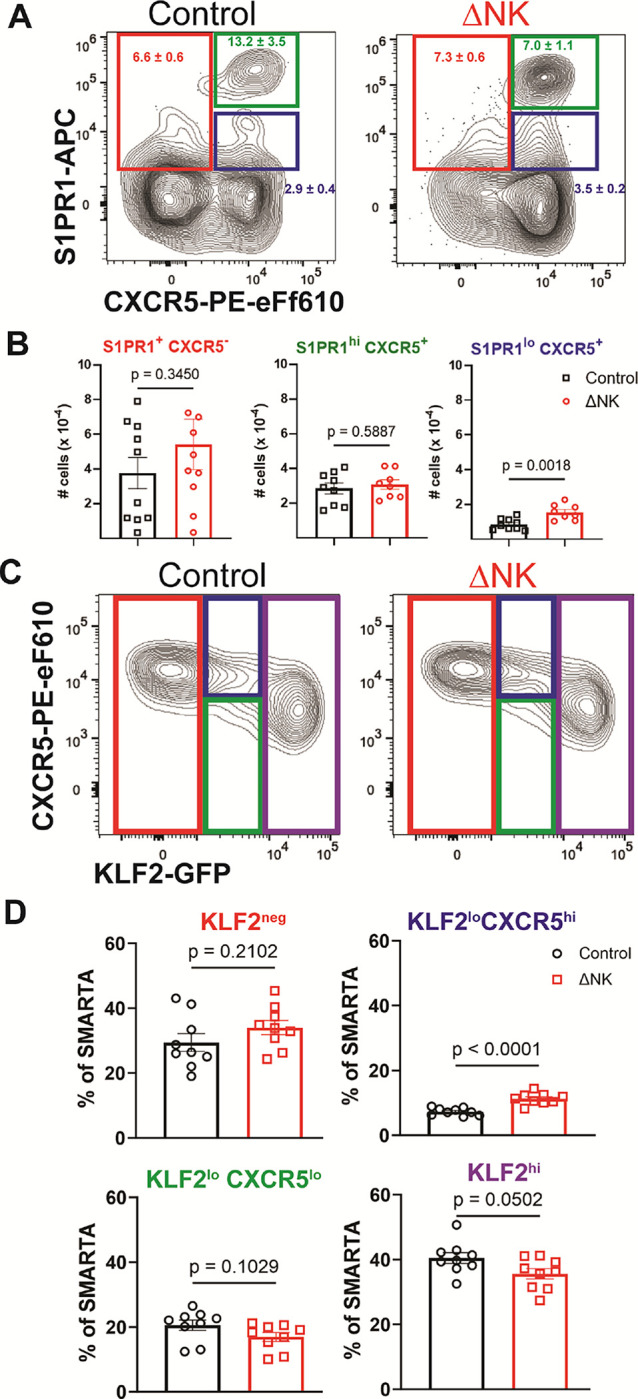
Protein confirmation of single cell RNA sequencing hits. **A)** Representative flow plots of T cells gated on live, CD4+ CD8-, CD45.1+, GP66–80+ SMARTA depicting the relationship between KLF2 and CXCR5 and enhancement of the CXCR5+ population within the KLF2 intermediate cells. **B)** Numerical depiction of the enhancement of the percent of SMARTA within that KLF2 low CXCR5 gate. **C)** Representative flow plot of the S1PR1 Intermediate cells. **D)** Frequency of SMARTA that fall into the gates in C.

To test this hypothesis, we first crossed *Klf2-GFP* transgenic mice to SMARTA mice to create a KLF2-GFP SMARTA mouse permitting indirect assessment of KLF2 expression levels. *Klf2-GFP* SMARTA cells were seeded into NK-cell depleted or control treated C57BL/6 prior to LCMV infection. At 84 hours p.i., responding donor cells were gated into CXCR5^+^ KLF2-GFP^low^ (Tfh), CXCR5^low^ KLF2-GFP^high^ (Th1), and those with intermediate expression of KLF2-GFP dividing into CXCR5 high and low subsets (Figure 4A). The presence of NK cells significantly reduced the proportions of Tfh-leaning (CXCR5^+^) cells with intermediate KLF2-GFP expression but not any of the other gated subpopulations of responding SMARTA (Figure 4B). We next focused on a canonical KLF2 target, sphingosine-1-phosphate receptor 1 (S1PR1), that was expressed at intermediate levels in the NK-cell susceptible cluster of developing Tfh cells (Figure 4C). Using flow cytometry, we found that the proportion and number of S1PR1^low^ CXCR5^+^ CD4 T cells were significantly repressed by NK cells while S1PR1^high^ CXCR5^+^ Tfh-like and S1PR1^+^ CXCR5^neg^ Th1-like cells were unaffected (Figure 4D). These results confirm the transcriptomic data by revealing intermediate proteomic expression of KLF2 and its target S1PR1 in developing Tfh is tightly linked to susceptibility of these cells to immunoregulatory functions of NK cells.

### KLF2+ SMARTA abundance is regulated by spatial localization.

**Figure 5:**
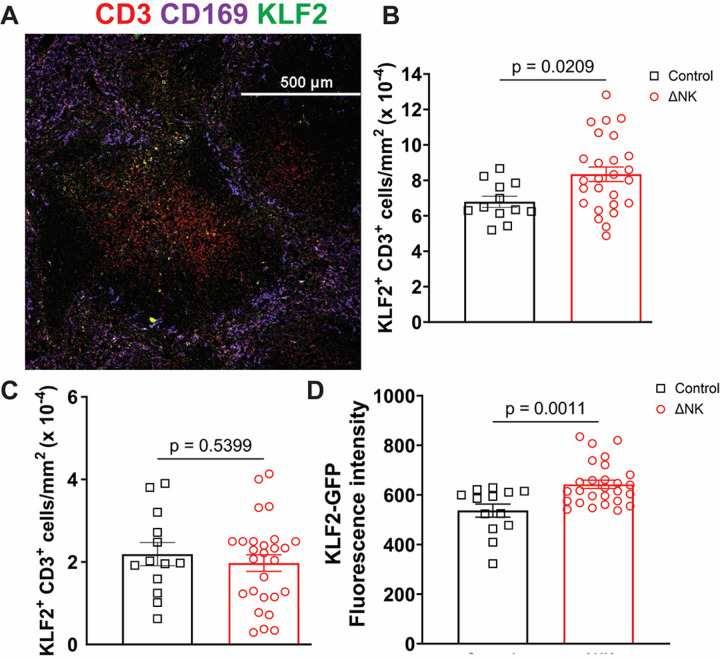
Spatial regulation of NK immunoregulation. KLF2-GFP SMARTA were transferred into B6 hosts as above and infected with LCMV. 84 hours spleens were harvested, fixed, dehydrated, frozen, cryosectioned and stained for CD3, CD169, DAPI and GFP. **A)** representative image. **B-C)** quantification of the number of KLF2-GFP + CD3 cells that are present within the **B)** white and **C)** red pulp respectively. **D)** MFI of KLF2-GFP within the T cells in the white pulp in NK depleted or isotype treated controls.

KLF2 dictates positioning of T cells within the spleen and other lymphoid organs.^20^ Since NK cells must migrate into the white pulp of the spleen to regulate activated CD4 T cells^21^, we reasoned that KLF2-expressing target T cells would be selectively reduced by NK cells in a spatially constrained manner. We quantified KLF2-GFP+ donor SMARTA cells within the white pulp (constrained by CD169+ marginal zone) or red pulp (outside marginal zone-constrained follicles) by microscopy at day 3.5 post infection (Figure 5A). An increased number of KLF2-GFP+ donor SMARTA T cells were found within the white pulp (Figure 5B) but not the red pulp (Figure 5C) of mice depleted of NK cells prior to infection in comparison to non-depleted controls. Moreover, depletion of NK cells increased the per cell intensity of KLF2-GFP expression in T cells (Figure 5D). These results highlight that spatial restrictions govern T cell susceptibility to the perforin-dependent immunosuppressive activities of NK cells.^21^

### NK-cell suppression requires KLF2 expression in CD4 T cells.

**Figure 6:**
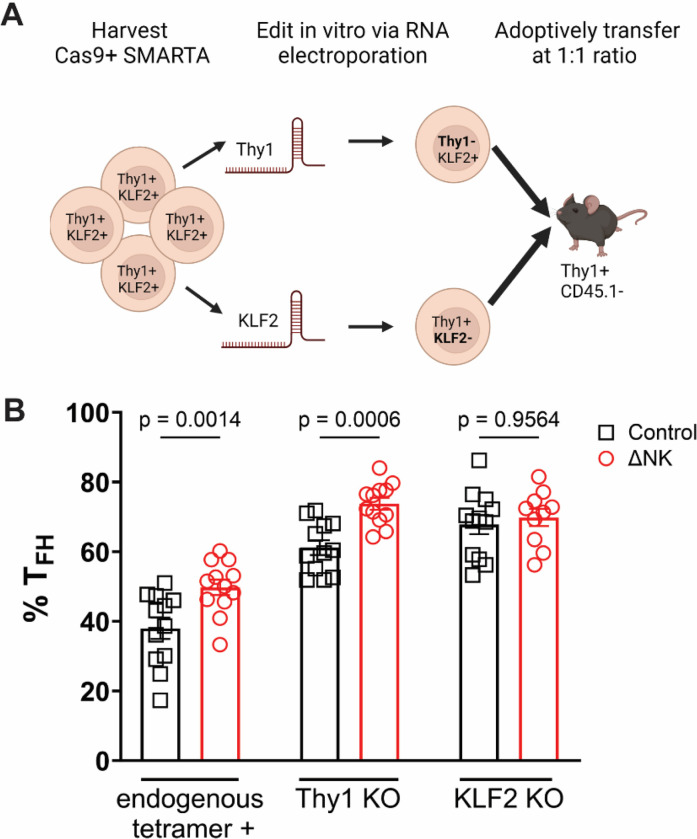
NK-cell suppression of antiviral Tfh responses depends on KLF2 in responding T cells. **A)** Simplified workflow of cell editing strategy. Cas9+ SMARTA CD4 T cells were electroporated with gRNAs targeting either *CD90* or *KLF2* and cultured in minimal cytokines for 6 days prior to adoptive transfer. We created an additional internal control through co-transfer of CD90 edited cells to account for within group variability. Mice were either depleted of NK cells or treated with isotype control antibody 1 day prior to adoptive transfer. The day following adoptive transfer mice were infected with LCMV Armstrong. We purposefully transferred in a smaller population of T cells than would be expected in the host precursor frequency and therefore harvested at a later timepoint to allow for robust SMARTA expansion. **B)** The frequency of Tfh is not altered by NK cell immunoregulation in KLF2 depleted SMARTA but is impacted in both the endogenous tetramer+ response as well as the CD90 edited SMARTA. Statistical analysis performed via One-way ANOVA. Depicted is the mean ± SEM.

To investigate the functional role of KLF2 in T cell susceptibility to immunoregulatory functions of NK cells, we generated Cas9-expressing SMARTA mice^22,23^ and transfected resulting CD4 T cells with small-guide RNAs specific for *Thy1* (Control) or *Klf2* (Figure 6A). We sequenced an aliquot of cells to verify gene targeting efficiency before co-transfer of equal numbers of these cells into C57BL/6 hosts that were depleted or not of NK cells prior to infection with LCMV Armstrong. As expected, NK-cell depletion skewed the phenotype of endogenous CD4 T cells and control (Thy1-edited) SMARTA cells towards Tfh lineages (Figure 6B **and gating strategy in Extended Data Figure 2**). Deletion of *Klf2* ameliorated this effect, with KLF2-deficient T cells exhibiting heightened skewing towards a Tfh phenotype that was not further affected by the depletion of NK cells (Figure 6B). Collectively, these findings establish the requirement of KLF2 expression in CD4 T cells to facilitate NK cell-mediated immunoregulation.

NK cell suppression of T cell responses has emerged as a critical determinant of vaccine efficacy and viral pathogenesis in both mice and humans.^1–5^ An improved understanding of the mechanisms by which NK cells recognize and are activated to kill target CD4 T cells would facilitate development of therapeutic methods to block this functionality to enhance vaccine efficacy. The results presented here cast doubt on the widely accepted belief that NK cell recognition and killing of CD4 T cells is mediated by cognate interactions between activating receptors and their ligands on target T cells. Instead, we demonstrate that susceptibility of CD4 T cells to NK cell suppression requires expression of the transcription factor KLF2. Correspondingly, activated CD4 T cells expressing intermediate levels of KLF2 and KLF2-target genes involved in spatial positioning are the predominant target of NK cells. Intermediate KLF2 expression on developing Tfh would position these cells at the T-B border where they are likely to spatially liaison with immunoregulatory NK cells infiltrating the T-cell zone.^21^ In addition, this positioning would lessen the inhibition of NK cells provided in trans by B cells expressing high levels of Qa1 (HLA-E).^11,12^ Thus, Tfh that are licensed to enter B-cell follicles are protected by surrounding B cells, leaving developing Tfh at the edge of the follicle susceptible to NK-cell regulation.^24^ NK cell culling of ‘excess’ or suboptimal Tfh remaining at the interface may help to prevent autoimmunity by controlling the quantity of activated T cells that gain access to potentially self-reactive B cells in an inflamed follicle.^25^ In summary, KLF2 control of spatial positioning of activated CD4 T cells during Tfh differentiation is a stronger determinant of susceptibility to NK cell-mediated immunoregulatory attack than expression of ligands for NK cell receptors. This represents a unique paradigm from which to consider development of strategies to enhance or subvert NK-cell suppression of T-cell dependent humoral immunity in the contexts of autoimmune disease or vaccination, respectively.

## Methods

### Mice:

C57BL/6, SMARTA LCMV-specific TCR transgenic mice, Perforin (Prf1) deficient, and Cas9-expressing mice were purchased from Jackson Laboratory (Bar Harbor, ME). KLF2-GFP mice were a gift from Sing Sing Way. Cas9 or KLF2-GFP mice were crossed to SMARTA and bred to homozygosity to create Cas9-SMARTA and KLF2-GFP SMARTA mice. Male mice between 8 to 20 weeks of age were routinely utilized in experiments. Mice were housed under barrier conditions and experiments performed under ethical guidelines approved by the Institutional Animal Care and Use Committees of Cincinnati Children’s Hospital Medical Center. Staff performing experimental measures were blinded to genotype and treatment status of experimental groups during sample processing and data acquisition-.

### Virus and viral vector injections:

Mice were infected with the Armstrong strain of LCMV via intraperitoneal injection of 5×10^4^ plaque forming units per mouse. Viral stocks were previously generated in house via propagation on BHK21 cells with titer determined using Vero cells.

### SMARTA transfer model:

SMARTA mouse spleens were harvested and processed into single cell suspensions (See Flow Cytometry) from which CD4 T cells were enriched by depletion of non-CD4 T cells (Miltenyi Biotec, Germany). 5×10^5^ CD4 T cells were injected retro-orbitally into isotype control and NK cell-depleted mice 1 day prior to LCMV infection. Two to four days following infection, spleens were harvested and subjected to flow cytometry or immunofluorescence. To ensure validity of the model an experiment was performed wherein groups of mice were treated as above but only 50,000 SMARTA were adoptively transferred. 4–7 days post infection the same trend of enrichment was seen in the SMARTA and endogenous T cell pools that recognize the MHC tetramer loaded with LCMV-GP_64–78_. This ensures that the supraphysiologic doses of SMARTA we used to detect this novel subpopulation behave similarly to a physiologic precursor frequency.

### In vivo NK-cell depletion:

One day before infection, selective depletion of NK cells^10^ was achieved through a single intraperitoneal injection of 25 micrograms of mouse anti-NK1.1 monoclonal antibody (PK136) or 25 micrograms of a control mouse IgG2a isotype antibody (C1.18.4) produced by Bio-X-Cell (West Lebanon, NH).

### Flow cytometry and in vitro peptide stimulation:

Single-cell leukocyte suspensions were prepared from spleens by mechanical homogenization of tissues between frosted glass microscope slides (VWR, Radnor, PA) and filtration through a 70 μm nylon mesh. Following lysis of red blood cells, lymphocytes were plated at 2×10^6^/well in 96-well round-bottom plates and subjected to flow staining. Leukocytes were washed with PBS then stained for dead cells with Zombie UV (BioLegend, San Diego, CA) used at 1:1000 for 5 min at room temperature, washed twice with PBS, and then Fc Receptor blocked with anti CD16/32 (BD BioSciences, San Jose, CA) used at 1:200 in FACS buffer (Phosphate buffer saline + 2% fetal bovine serum + 0.5 mM EDTA) for 5 min at 4C prior to surface staining with the following antibodies: CD162 (Clone 2PH1 used at 1:300), CD8α (Clone 53–6.7 1:200), CD45R (Clone RA3–6B2 used at 1:200), CD4 (Clone GK1.5 used at 1:200), SLAMF6 (Clone 13G3 used at 1:200), Ly6c (HK1.4 used at1:300), CD69 (Clone H1.2F3 used at 1:200), Integrin ß7 (Clone FIB504 used at 1:100), GP64 tetramer (Obtained from the NIH 1:75), CXCR5 (Clone SPRCL5 used at 1:100), S1PR1 (clone MAB7089 used at 1:50), SLAMF1 (Clone TC15–12F12.2 used at 1:100), CD19 (Clone 6D5 used at 1:200), NK1.1 (Clone PK136 used at 1:100), CD49b (Clone DX5 used at 1:100), CD45.1 (Clone A20 used at 1:100), CD62L (Clone MEL-14 used at 1:200). All antibodies were purchased from BioLegend (San Diego, CA), BD Biosciences (San Jose, CA), or ThermoFisher Scientific (Waltham, MA). GP64 loaded MHCII tetramers and S1PR1 stains were used at 37C for 90 min prior to addition of the remaining antibodies in Brilliant Stain buffer and room temperature incubation for 30 minutes. Following staining, cells were washed and fixed with BD fixation buffer (BD Biosciences, San Jose, CA) for 5 minutes at 4°C. For experiments utilizing KLF2-GFP expressing mice flow cytometry was run on the same day as harvest without fixation to preserve GFP signal. Cells were washed twice in FACS and resuspended in 100 microL FACS buffer and run on a Cytek Aurora Spectral flow cytometer. Spectral unmixing was performed in SpectroFlow and flow plots created using FlowJo.

### Single cell RNA sequencing:

GP66–81:I-A^b+^ CD45.1^+^ cells were sorted from LCMV infected animals, loaded onto the Chromium platform (10 X Genomics) to generate cDNAs carrying cell- and transcript-specific barcodes that were used to construct sequencing libraries using the Chromium Single cell 5’v2 Library & Gel Bead Kit according to the manufacturer instructions. Libraries were sequencing on a single run of Illumina Novaseq 6000 using paired-end reads to reach a read depth of 28–30,000 reads per cell. Raw base call files were de-multiplexed with Cell Ranger ^26^ v3.0.2 mkfastq (10x Genomics). Reads were aligned to mouse reference genome mm10 and gene expression quantified using Cell Ranger count. Further data analysis was carried out with Seurat ^27,28^v4.3.0.1 in R v4.2 ^29^. Cells displaying more than 5% mitochondrial gene expression, less than 500 total expressed genes, or less than 1000 RNA counts were excluded from the analysis. Gene expression counts were normalized with the NormalizeData function in Seurat, which uses a logarithmic normalization method where gene counts for each cell are divided by its total counts and natural log-transformed using log1p and multiplied by a scale factor of 10,000. Data was scaled using the ScaleData function in Seurat and mitochondrial score and cell cycle genes were regressed out of the data. Doublets were removed using doubletFinder^30^. The six samples were integrated together using FindIntegrationAnchors and IntegrateData functions from Seurat. This integrated dataset was used for principal component analysis, variable gene identification, Shared Nearest Neighbor (SNN) clustering analysis, and Uniform Manifold Approximation and Projection (UMAP). Clusters were defined to optimize silhouette scoring. DotPlot of common CD4 T cell defining genes shown in Supplemental Figure 3. Clusters were collapsed for each individual animal for pseudobulk analysis using AggregateExpression and compared using DESeq2.

### Tissue processing, sectioning, and immunohistochemistry:

Tissues to be analyzed by fluorescence microscopy were placed into 4% formaldehyde solution for 5 hours followed by overnight dehydration in a 30% sucrose (Sigma-Aldrich, St. Louis, MO) solution at 4°C. Samples were washed with phosphate buffered saline prior to embedding within optimal cutting temperature (OCT) media (Sakura Finetek, Maumee, OH) and frozen using a dry ice slurry in 100% ethanol and stored in −20°C. Tissues were sectioned (7–10 μm thick) using a cryostat (Leica CM3050 S) and affixed to positively charged Denville slides (Thomas Scientific, Swedesboro, NJ). Slides were dried at room temperature for 5 to 10 minutes preceding a 10-minute fixation in chilled 100% acetone at −20°C. Slides were subsequently dried at room temperature for 5 to 10 minutes and washed twice in chilled phosphate-buffered saline before being placed in saturation buffer containing 10% normal donkey serum (Sigma-Aldrich, St. Louis, MO) and 0.1% Triton-X (Sigma-Aldrich, St. Louis, MO) in phosphate buffered saline for blocking at room temperature for 45 minutes. Slides were incubated with a primary antibody cocktail containing one or more of the following antibodies overnight at 4°C: 1:100 goat anti-NKp46 (R&D Systems, Minneapolis, MN), 1:200 rat anti-CD3e-AF647 (Clone 17A2 from R&D Systems, Minneapolis, MN), 1:200 rat anti-CD169-AF594 (3D6.112, BioLegend, San Diego, CA) 1:200 anti-CD45R/B220-AF657 (Clone RA3–6B2 from ThermoFisher Scientific, Waltham, MA), and 1:100 chicken anti-GFP (Aves). The following day, slides were washed twice in chilled phosphate buffered saline. Subsequent secondary antibody staining with 1:1000 donkey anti-goat AF555 (ThermoFisher Scientific, Waltham, MA) for 2 hours at room temperature to reveal NKp46 primary staining. Slides were again washed thrice with chilled phosphate buffered saline and additional secondary staining was performed with 1:5000 donkey anti-chicken AF488 (ThermoFisher Scientific, Waltham, MA) for an additional 2 hours at room temperature to reveal GFP primary staining. Slides were again washed twice with chilled phosphate buffered saline, mounted with prolong diamond mounting media (Thermo-Fisher Scientific, Waltham, MA), covered with a coverslip, and allowed to cure overnight prior to imaging.

### Confocal microscopy & NK-cell quantification:

Confocal imaging was performed using a Laser Scanning Nikon AXR Inverted Confocal Microscope with NIS Elements Confocal software. Z-stacked tissue images were acquired through a 20X objective (Nikon Plan Apo λ) from which a maximum intensity projection was generated prior to cell enumeration. Using tools available in NIS Elements Analysis software and guided by CD169- and CD3-staining, borders around white pulp (CD169 boundary) and T cell zones (CD3 boundary) were drawn. To enumerate KLF2-GFP+ CD3+ T cells, a cell expansion algorithm was used to determine the number of GFP and CD3 positive cells (cells borders determined by DAPI staining). Brightness and contrast for each representative image were adjusted equally across all channels using Photoshop CS6.

### Statistics:

Experimental results are consistently presented as the mean with individual data point spread. Statistical differences between control and experimental groups were calculated using a one-way analysis of variance (ANOVA) with either the Holm-Šídák multiple comparison test or a Dunnett’s multiple comparisons test. A p-value of less than 0.05 was considered significant. Graphing and statistical analysis were routinely performed using GraphPad Prism (San Diego, CA). Researchers were blinded to groupings and treatment during experimental measurements.

## Limitations

The studies herein employed transgenic T cells in the LCMV infection model with accompanying strengths and limitations. As we desired to examine these very early time points, we utilized transgenic cells to query subpopulations as we could adoptively transfer a large initial population of antigenic specific T cells. We validated this approach by looking for the Tfh/Th1 skewing of the endogenous population at various times p.i. and we confirmed that the response of the endogenous T cells that recognize the MHC tetramer loaded with LCMV-GP64–78 mimics the SMARTA response (**Extended Data Figure 3**). Additionally, we used LCMV as it is the strongest, but not only, inducer of NK immunoregulation. We and others have shown that many other infections and vaccination modalities induce NK immunoregulation^6–8^ so we expect our results to be generalizable to additional inflammatory stimuli.
